# *MGMT* methylation pattern of long-term and short-term survivors of glioblastoma reveals CpGs of the enhancer region to be of high prognostic value

**DOI:** 10.1186/s40478-023-01622-w

**Published:** 2023-08-28

**Authors:** Henning Leske, Ulrike Camenisch Gross, Silvia Hofer, Marian Christoph Neidert, Sabine Leske, Michael Weller, Dirk Lehnick, Elisabeth Jane Rushing

**Affiliations:** 1 Department of Pathology, Oslo University Hospital, Oslo, Norway; 2 University of Oslo (UiO), Oslo, Norway; 3 Department of Neuropathology, University Hospital of Zurich, Zurich, Switzerland; 4 Department of Pathology, University Hospital of Zurich, Zurich, Switzerland; 5 Department of Neurology, University Hospital and University of Zurich, Zurich, Switzerland; 6 Department of Neurosurgery, University Hospital of Zurich, Zurich, Switzerland; 7 Department of Neurosurgery, Cantonal Hospital of St. Gallen, St. Gallen, Switzerland; 8 Department of Health Sciences and Medicine, Head Biostatistics and Methodology, University of Lucerne, Lucerne, Switzerland; 9 Department of Pathology/ Neuropathology, Lucerne, Switzerland

**Keywords:** Glioblastoma, *MGMT*, Survival, Enhancer, Prognosis, Temozolomide

## Abstract

Treatment with the alkylating agent temozolomide is known to be prognostically beneficial in a subset of glioblastoma patients. Response to such chemotherapeutic treatment and the prognostic benefit have been linked to the methylation status of *O*^*6*^*-methylguanine-DNA methyltransferase* (*MGMT*). To date, it has not been entirely resolved which methylation pattern of *MGMT* is most relevant to predict response to temozolomide treatment and outcome. In this retrospective study, we compared the methylation patterns, analyzed by Sanger sequencing, of 27 *isocitrate dehydrogenase* (*IDH*)-wildtype glioblastoma patients that survived more than 3 years (long-term survivors) with those of 24 patients who survived less than a year after initial surgery (short-term survivors). Random Forest-, Correlation-, and ROC-curve analyses were performed. The data showed that *MGMT* is typically methylated in long-term survivors, whereas no prominent methylation is observed in short-term survivors. The methylation status of CpGs, especially in the promoter and exon1/enhancer region correlated highly with outcome. In addition, age and temozolomide treatment were strongly associated with overall survival. Some CpGs in the enhancer region, in particular CpG 86 (bp + 154), demonstrated high values associated with overall survival in the Random Forest analysis. Our data confirm previously published prognostic factors in *IDH*-wildtype glioblastoma patients, including age and temozolomide treatment as well as the global *MGMT* methylation status. The area frequently used for decision making to administer temozolomide at the end of exon1 of *MGMT*, was associated with outcome. However, our data also suggest that the enhancer region, especially CpG 86 (bp + 154) is of strong prognostic value. Therefore, we propose further investigation of the enhancer region in a large prospective study in order to confirm our findings, which might result in an optimized prediction of survival in glioblastoma patients, likely linked to response to temozolomide treatment.

**Supplementary Information:**

The online version contains supplementary material available at 10.1186/s40478-023-01622-w.

## Introduction

Glioblastomas are the most common primary brain tumors in adults with a median overall survival (OS) of 12 months at the population level [26]. Still, there are patients who survive more than 3 years [6, 10]. Epigenetic alterations of the gene *MGMT* encoding the DNA repair protein O^6^-methylguanine-DNA methyltransferase have been found to be of prognostic and predictive relevance for chemotherapeutic response to alkylating agents such as temozolomide [4, 9, 19]. Patients with *MGMT* promoter-methylated tumors have been found to be overrepresented in long-term survivors [8, 12].

The CG-rich dinucleotide sequence (CpG-island) of *MGMT*, which contains 98 CpGs [18, 25], encompasses the promoter, minimal promoter, exon 1, enhancer and intron 1 region. Several studies have shown that the methylation status of CpGs within this CpG-island is critical for MGMT expression, and thus patients with methylated *MGMT* benefit from temozolomide treatment [17]. Standard methods for determining the *MGMT* methylation status comprise methylation specific PCR, pyrosequencing and DNA methylation microarrays [1, 9, 17]. The widely used MGMT-kit from Therascreen uses pyrosequencing to analyze the methylation status of CpGs 79–82 [13]. Although these results are used to stratify patients for temozolomide treatment, a consensus is lacking on which CpG sites and the corresponding cut-off levels are of prognostic relevance [22]. Only single studies have systematically investigated the methylation status of larger areas of *MGMT* in glioblastoma specimens [17, 23]. Furthermore, most frequently used methods (i.e. Therascreen, Epic array) analyze only a limited number of CpGs, which might not be sufficient to predict response to temozolomide treatment.

In the current retrospective study, we compared the *MGMT-*methylation pattern of *isocitrate dehydrogenase* (*IDH*)-wildtype glioblastomas from patients, who survived more than 3 years (long-term survivors) with those who survived less than 1 year (short-term survivors). Specifically, we analyzed 79 CpGs, covering the previously deemed most relevant sites [18] for *MGMT*-silencing, on bisulfite converted DNA using methylation independent PCR followed by Sanger sequencing. The aim was to systematically investigate *MGMT* methylation patterns that correlate with outcome and the likely related benefit from temozolomide treatment.

## Material and methods

### Patients

Patients with *IDH*-wildtype glioblastoma or histological variants, e.g., gliosarcoma, diagnosed after 2001 with a follow-up until May 2016, were included in this study. The study cohort comprises 32 long-term survivors, defined as patients with an overall survival longer than three years after initial diagnosis, and 25 short-term survivors, where overall survival was less than one year. Of those, five long-term survivors and one short-term survivor had to be excluded due to insufficient DNA quality, resulting in 27 long-term survivors and 24 short-term survivors. Two neuropathologists (HL and ER) confirmed the diagnoses. The following clinical data were assessed: age at diagnosis, sex, tumor localization, temozolomide administration, type of adjuvant therapy and overall survival (OS).

### Molecular analyses

All cases were investigated using immunohistochemistry for IDH1 p.R132H, ATRX expression and nuclear p53 accumulation. In cases where nuclear ATRX expression was technically inconclusive, *IDH*-sequence alteration status was reinvestigated at codon 132 of *IDH1* and codon 172 of *IDH2* by Sanger sequencing (for further information see Additional file 1:supplementary materials and methods) [15].

*MGMT* analysis was performed on bisulfite converted DNA using methylation independent PCR followed by Sanger sequencing. The location of the CpG site was defined based on the description of the CpG-island of *MGMT* ranging from base pair (bp) − 552 to + 289 [18]. In this study we analyzed all CpG sites starting from CpG 23 (bp − 300) to the end of the CpG island (bp + 289), as well as the three subsequent CpG sites (bp + 292, + 296, + 309). For methylation independent PCR covering the investigated area of DNA, the three primer pairs PROM (CpG 23 to 62), E1I1 (CpG 63 to 101) and ATG1 (CpG 75 to 101) were designed. Methylation levels ranged from 5%, which was defined as the lowest value for unmethylated sites, to 100% and were calculated in relation to a tonsil control (for further information see Additional file 1: supplementary materials and methods).

### Ethical statement

This study was conducted according to the ethical principles of medical research involving human subjects according to the Declaration of Helsinki. The clinical data were assessed and anonymized for patients’ confidentiality. All patients that were alive at the time of the analysis provided written informed consent. According to the Swiss ordinance on Human Research with the Exception of Clinical Trials (HFV) such consent was not needed for patients that were already deceased at the time of the analysis, provided that the cases were anonymized, that there was no documentation that their specimen should not be used for research, that the research is in the interest of other patients suffering from the disease and that it is impossible or disproportionally difficult to obtain inform consent from these patients or their relatives. The study was approved by the Ethics Committee of the Canton of Zurich (KEK-ZHNr.2013-0035).

### Statistical analyses

For analysis of discontinuous survival data Random Forest regressions were performed. It was calculated to predict patient survival based on the percentage of CpG methylation for CpG sites 23–101 (79 in total), which were used as predictors. The regression model aggregated the results of 1000 regression trees and at each split, the algorithm considered m_try_ = 35 predictors to find the best split.

To additionally include categorical predictors such as age, temozolomide treatment, brain lobe and hemisphere, a Conditional Random Forest analysis, using similar parameters as for Random Forest analysis, was performed to avoid bias towards continuous variables. This algorithm provides a respective conditional variable importance. Since Conditional Random Forest algorithm requires a complete data set without missing values, values that could not be determined by PCR were imputed using the MissForest algorithm (See Additional file 5: Table ST1), which was applied to the predictor values of all patients excluding the dependent variable survival. Random Forest analyses were performed using R version 3.5.0 with the packages “party” version 1.3-1 and “missForest” version 1.4.

An important feature of Random Forest is the Variable Importance for each predictor, which indicates how important and informative the predictor was for the splitting and prediction of the Random Forest trees [27]. For the respective estimation the Permutation Accuracy Importance was used, which compares the actual prediction error with the prediction error while a predictor variable is randomly permuted [24]. The conditional variable importance was used, which indicates the importance of each predictor considering the influence of all other predictors in the model. Therefore, the measure corrects for the intercorrelation of the predictor variables [24].

In order to evaluate the performance of the Random Forest regression models, a pseudo R^2^ according to Grömping (Grömping, 2009) was calculated to estimate the amount variance explained by the model with the following formula: OOB-*R*^2^ = (1 – MSE/SST), where MSE stands for Mean Squared Error, which is calculated with the help of the Out Of Bag-data (OOB-Data), and SST stands for Sum of Squares Total. Methylation data were visualized using Graphpad Prism 6.0 and the correlation plot was performed using Matlab.

ROC-curve analyses were performed using STATA (Version 16.0 or later, StataCorp, College Station, Texas, USA). Only CpG sites 75–101 were investigated using the data set containing MissForest imputed values. Survival of patients was dichotomized and grouped into long-term and short-term survivors. ROC analyses have been performed for either individual CpGs or for combinations of four CpG sites.

## Results

### Patient characteristics and *MGMT* methylation pattern of long-term and short-term survivors

Data reveals heterogeneity among long-term and short-term survivors. Gender was distributed equally in the group of short-term survivors, whereas two thirds of long-term survivors were male. Long-term survivors were younger at time of initial surgery (median age at diagnosis 50.5 years) than short-term survivors (median age at diagnosis 70.0 years). In addition, localization of the tumor was more often frontal (44%) in long-term survivors, whereas parietal lobes were more often affected in short-term survivors (46%). All neoplasms of short-term survivors were classified as glioblastoma (*n* = 24). However, long-term survivors contained glioblastomas and histological variants with 81% glioblastoma (*n* = 22), 11% gliosarcoma (*n* = 3), 4% giant cell glioblastoma (*n* = 1) as well as 4% epithelioid glioblastoma (*n* = 1). All long-term survivors received temozolomide treatment, whereas only 46% of the patients in the short-term survivor cohort received this drug (Fisher’s exact test: *p* < 0.001) (Table 1).

**Table 1:** Patient characteristics of long-term and short-term survivors

		Long-term survivor	Short-term survivor
Sex	Male	18 (67%)	12 (50%)
	Female	9 (33%)	12 (50%)
Median age at diagnosis in years (range)		50.5 (34.0–68.1)	70.0 (35.7–83.9)
Median follow-up time in years (range)		4.8 (3.1–8.6)	0.4 (0.0–0.9)
Vital status at end of individual follow-up	Alive	10 (37%)	0 (0%)
	Dead	17 (63%)	24 (100%)
Subentity	Glioblastoma	22 (81%)	24 (100%)
	Giant cell glioblastoma	1 (4%)	0
	Gliosarcoma	3 (11%)	0
	Epithelioid glioblastoma	1 (4%)	0
Tumor location	Frontal	12 (44%)	2 (8%)
	Temporal	7 (26%)	9 (38%)
	Parietal	5 (19%)	11 (46%)
	Occipital	3 (11%)	2 (8%)
Laterality	Left	14 (52%)	12 (50%)
	Right	13 (48%)	12 50%)
Temozolomide administration		27 (100%)	11 (46%)

Comparison of features from long-term (more than 3 years overall survival) and short-term survivors (less than 1 year overall survival) are depicted based on sex, median age at initial diagnosis and median follow-up time in years, individual vital status at the end of the follow-up (May 2016), tumor location, subentity and temozolomide administration

Temozolomide administration was associated with longer survival within the group of short-term survivors (median survival 7.9 vs. 2.9 months; log-rank test: *p* = 0.001).

### *MGMT* methylation pattern of long-term and short-term survivors

In a subset of cases, the DNA quality was limited resulting in missing values at some CpG sites. Most missing values were associated with analyses using the E1I1 primer pair. Further analyses of areas covered by E1I1 and ATG1 are therefore depicted based on the data generated with the ATG1 primer pair (i.e. CpGs 75–101) (Additional file 6: Table ST1 and Additional file 2: Fig. S1).

**Fig. 1:**
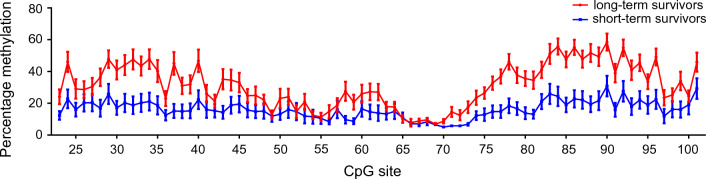
Methylation pattern of long-term and short-term survivors of glioblastoma. The mean ± SEM of relative percentage of methylation from all long-term and all short-term survivors of glioblastoma per CpG of *MGMT* reveals differences between these groups. Whereas glioblastoma samples of long-term survivors demonstrate frequently methylation in areas CpGs 28–40 and CpGs 75–96, those areas in neoplasms of short-term survivors are mainly unmethylated. The connecting line is added for visualization purposes

The obtained data illustrate that short-term survivors show rather low levels of CpG-methylation within the analyzed *MGMT* region. In contrast, tumors from long-term survivors often displayed increased CpG-methylation with two main peaks: The first peak covers CpGs 28–40 and the second peak encompasses CpGs 75–96 (Fig. 1).

Additionally, in 2 long-term survivors, where both, the initial specimen and the recurrence tissue has been analyzed, there were major differences of the methylation of several CpGs of *MGMT* observed. While particularly CpGs 75–101 and partially CpGs 23–59 of the initial specimen was methylated, methylation was almost completely lost in tumor recurrences after 3 and 7 years, respectively (Additional file 3: Fig. S2).

### *MGMT* methylation pattern of CpGs 23-101

**Fig. 2:**
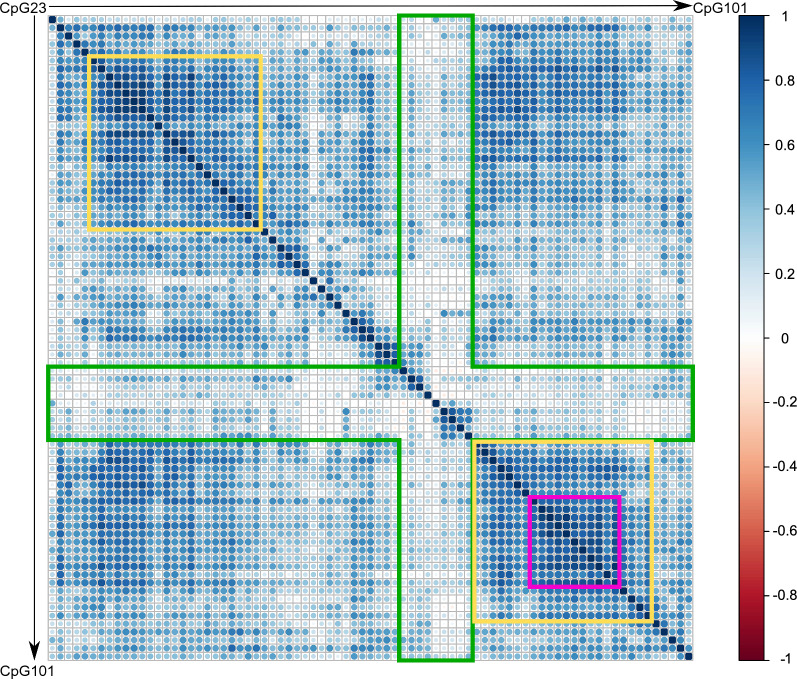
Correlation matrix including all analyzed CpGs of *MGMT.* The correlation plot reveals that several CpG sites within the analyzed area, ranging from CpG 23 (bp -300) to CpG 101 (bp + 309), are strongly correlated. Two clusters (highlighted in yellow) with increased correlation are seen; (CpGs 28–48) in the promoter region and (CpGs 75–96) encompassing exon1, enhancer and the intron1 region. In the latter, a cluster with very strong correlation (CpGs 82–92) emerged (highlighted in pink). In turn, CpGs of exon 1, after the minimal promoter (CpGs 66–74) with highest percentage of missing values (Additional file 2: Fig. S1) and lowest level of methylation (see Fig. 1), demonstrated less correlation (highlighted in green)

For further characterization of the CpGs` methylation, we investigated the correlation between individual CpG sites of the analyzed *MGMT* region. The correlation matrix (Fig. 2) revealed that there is a high correlation between CpGs 28–48 and CpGs 75–96 (highlighted in the yellow boxes). Here, particularly CpGs 82–92 (highlighted in the pink box) were most prominently correlated. In turn, the region CpG 66–74, which also showed the lowest level of methylation (Fig. 1) and the highest percentage of missing values (Additional file 2: Fig. S1), appeared to be the least correlated area (highlighted in the green surrounded area).

**Fig. 3:**
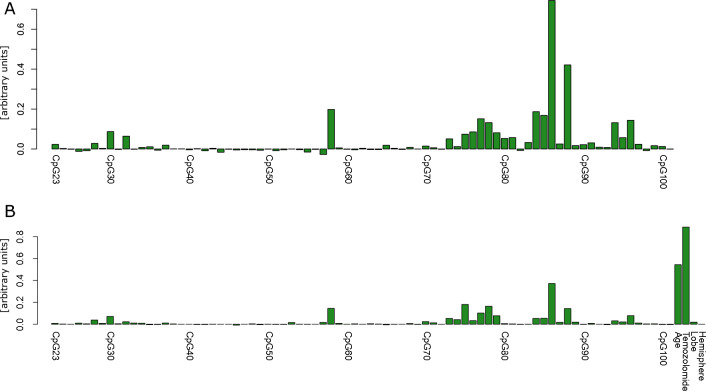
Random Forest analysis of all CpGs. Random Forest analysis demonstrates that CpG 86 has the highest predictive value of all tested CpGs. **A** Analysis of the raw data highlights CpGs 58, 73–79, 84–88 and 94–96 to be correlated with overall survival. Here, CpG 86 (bp + 154) demonstrate by far the most prominent value, followed by CpG 88 (bp + 180). **B** For additional inclusion of other predictive factors, such as temozolomide treatment, age at diagnosis, affected lobe and hemisphere, missing values had to be imputed to perform Conditional Random Forest analysis. Conditional Random Forest valued temozolomide administration and age of the patient as the strongest predictor of outcome followed by two clusters CpG 73–79 and CpG 84–88, which were similar to those with the raw data **A**. Also within the analysis with imputed data the methylation state of CpG 86 (bp + 154) resulted in the highest predictive value among all analyzed CpGs of *MGMT*

Next, we explored the potential of all tested CpGs to predict glioblastoma patient survival using the multivariate non-parametric method Random Forest regression. Analysis of the raw data (Fig. 3A) revealed that CpG 86 (bp + 154) was the site with the highest variable importance, a measure provided by Random Forest to indicate the informational value for the regression model and the predictor importance. Despite CpG 58 (bp -37), which showed the third highest predictor importance in this analysis, several sites of the enhancer region CpG 84–86 (bp + 143  –  bp + 154) and CpG 88 (bp + 180) where among those with the highest values, followed by the CpGs 75–81 (bp + 94 – bp + 126), CpGs 94 (bp + 242) and 96 (bp + 256). The estimated Pseudo-R^2^ (also referred to as OOB-R^2^) of the regression model according to Grömping (2009), (Grömping, 2009) was 0.5257. Accordingly, the Random Forest regression model explained 52.57% of variance of overall glioblastoma patient survival. We additionally included other factors, such as age, temozolomide treatment and location of the tumor, which were previously published as being associated with outcome in glioblastoma patients [2, 14]. Therefore, we performed multivariate testing using the Conditional Random Forest analysis.

The Conditional Random Forest analysis (Fig. 3B) revealed that temozolomide treatment and age at diagnosis show the highest conditional variable importance values with respect to the prediction of outcome of the patients. In addition, this analysis mostly confirmed the results on the CpG sites from the non-imputed data (Fig. 3A) with the highest values being CpGs 58 (bp -37), 73–79 (bp + 70 –  bp + 119), 84–88 (bp + 143 – bp + 180) and 96 (bp + 256). Tumor location proved to be of minor importance. Also in this analysis CpG 86 (bp + 154) demonstrated the highest predictor importance among CpG sites. The Conditional Random Forest regression model explained 60% of variance of overall glioblastoma patient survival. As mentioned above, these results included either missing values (Fig. 3A) or MissForest based imputed data (Fig. 3B) at sites where no methylation level could be analyzed due to low DNA quality.

### *MGMT* methylation pattern of CpGs 75-101

To avoid major influences based on data imputation on the results, we further focused on the area covered by the ATG1 primer pair for statistical analyses. For this region we were able to obtain the methylation level of all covered CpG sites, i.e., CpGs 75–101 (bp + 94 – bp + 309), except for 0.15% missing values (2 data points) (Additional file 2: Fig. S1).

The correlation matrix of methylation data from all patients, in addition to age and survival, revealed the correlation between different CpGs (Additional file 4: Fig. S3), which was mostly similar to the correlation over all CpGs as depicted in Fig. 2.

**Fig. 4:**
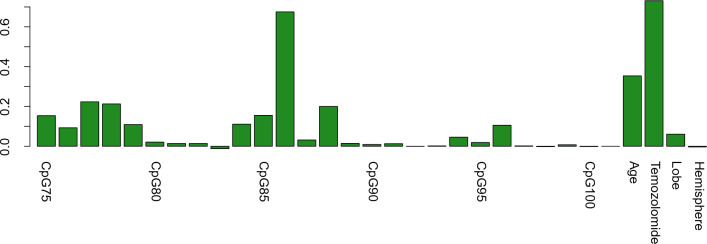
Conditional Random Forest of CpGs 75–101 of *MGMT.* Conditional Random Forest analysis including CpGs 75–101 as well as temozolomide, age at diagnosis, lobe and hemisphere. Random Forest analysis including imputed data revealed increased signals in the area of CpGs 75–79, CpGs 84–88 as well as at CpGs 94–96. Temozolomide treatment was the factor with the highest signal and, therefore, demonstrated the strongest correlation with outcome. CpG 86 (bp + 154) showed the second highest value followed by age at diagnosis

Random Forest analysis from the CpGs 75–101 revealed that specifically CpG site 86 (bp + 154) had by far the highest predictor importance value of all CpGs investigated, which was slightly below the value of temozolomide administration but higher than the value of age of the patients (Fig. 4). CpG predictor importance values show mainly two peaks. The first peak ranges from CpGs 75–79 and the second peak covers the area of CpGs 84–88. A small third cluster of increased values is present at CpGs 94–96.

### ROC-curve analysis

We further investigated CpG sites based on Random Forest results and correlation matrix to search for other CpG combinations that might correlate with increased overall survival. For the two missing values within our data set, we used the imputed data from the MissForest analysis (Additional file 6: Table ST1). Besides the CpG combination measured by Therascreen (CpGs 79–82) (MC1), we analyzed the combination of CpGs 77, 78, 86, 88 (MC2); CpGs 78, 80, 86, 96 (MC3) and CpGs 75, 78, 86, 96 (MC4).

MC2 was selected based on the CpG sites with the highest single values in the Random Forest analysis. In MC3, the CpG site 77, which is highly correlated with CpG 78 has been exchanged to the CpG site 80, which showed a good area under the curve when calculating ROC-curves for single sites (Additional file 5: Fig. S4).

In addition, CpG site 88, which is strongly correlated with CpG 86, has been exchanged with CpG 96, which shows a higher signal in the Random Forest analysis and is less correlated with other sites. In MC4, CpG 80 has been exchanged with CpG 75, which is one of the CpG sites with the lowest correlation in the area covered by the ATG1 primer pair (Fig. 2).

**Fig. 5:**
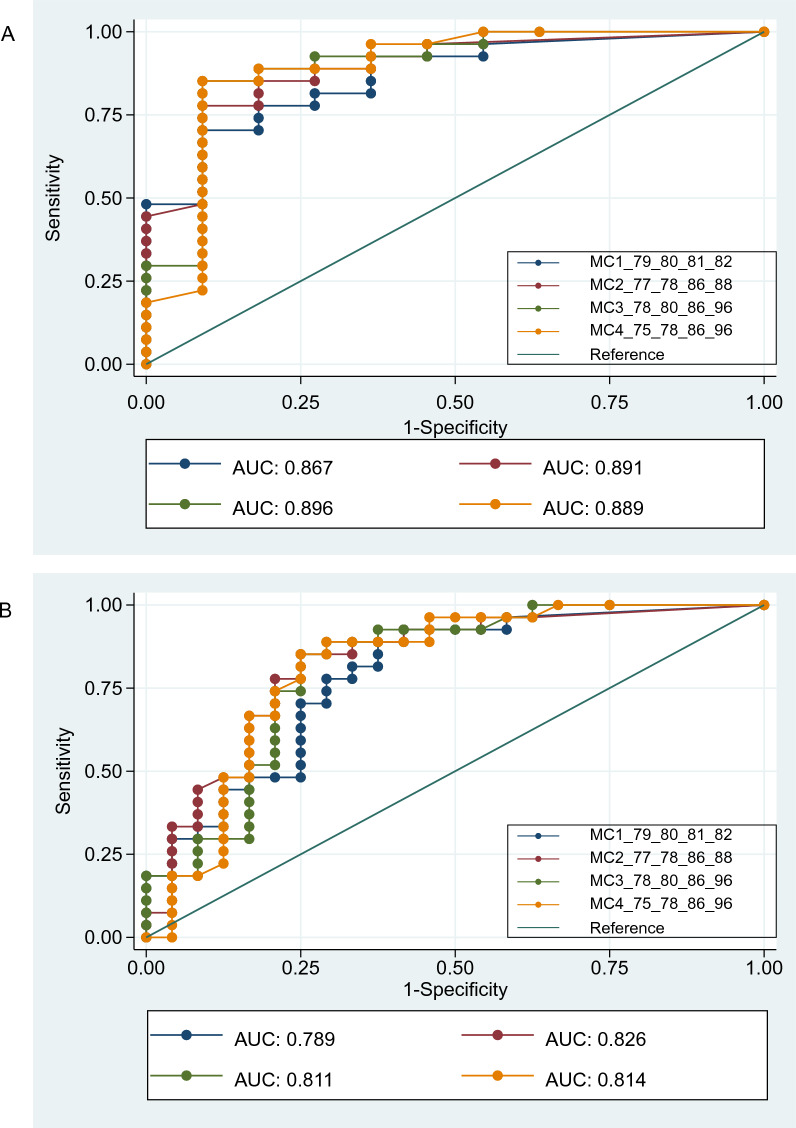
ROC-curves. Four combinations of CpG sites were investigated. MC1 was chosen according to the CpG sites that are commonly analyzed by the Therascreen pyrosequencing kit and often used in clinical practice. The other CpG site combinations MC2-MC4 were chosen based on features observed in the correlation analysis, Random Forest analysis and ROC analysis for single CpGs. The 2 missing values in the data set were imputed based on MissForest algorithm. In **A** only patients treated with temozolomide were investigated. No major changes in the area under the curve (AUC) were observed, ranging from 0.867 (MC1) to 0.896 (MC3). In **B **all patients from this cohort were investigated. Also here there were no major differences observed ranging from MC1 with an AUC of 0.786 to MC2 with an AUC of 0.826

ROC-curve analysis revealed no major differences between CpGs 79–82 and the other CpG combinations analyzed. Figure 5A demonstrates all patients that were treated with temozolomide. No major changes in the area under the curve (AUC) were observed, ranging from 0.867 (MC1) to 0.896 (MC3). Figure 5B shows all patients independently from temozolomide treatment. Also here, there were no major differences observed with the CpG combination MC1 demonstrating again an only mildly lower AUC of 0.786, whereas this time the MC2 CpG combination showed the highest AUC of 0.826.

Furthermore, these ROC-curves revealed that a methylation of 20% of the CpG combination (MC1), was sufficient to be associated with a predictive value, whereas the cut-off of the CpG combination (MC2-4) was about 25% and thus slightly higher.

## Discussion

The relationship between *MGMT* methylation and increased overall survival, possibly due to response to temozolomide treatment, is of major interest to clinicians dealing with glioblastoma patients. Several prospective studies have examined subsets of CpGs of *MGMT* [3] in high-grade gliomas, however, to date only few have systematically investigated a large number or even all CpG sites of the *MGMT* CpG-island [17]. In contrast to previous studies, we approached this question from a different angle. We selected *IDH*-wildtype glioblastomas from patients who either survived longer than 3 years or died within the first year after initial diagnosis and compared the *MGMT* methylation pattern of these tumors. The strategy was to focus on overall survival, while looking for specific methylation patterns that might correlate with patient outcome.

**Fig. 6:**
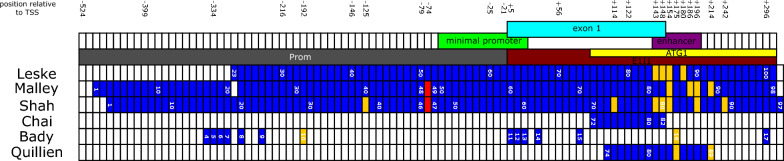
Analyzed CpGs of *MGMT* and correlation with other studies. The analyzed CpGs of the current study starting from CpG 23 (bp -300) to CpG 101 (bp + 309) and selected previous studies are highlighted in blue. The relative position to the transcription start site (TSS) are given. Primers that cover the area included in this study comprise Prom (gray), E1I1 (brown), ATG1 (yellow). Regions of *MGMT* are depicted as follows: minimal promoter (light green), exon1 (turquoise), enhancer (pink) CpG site 51, which has been disregarded by some authors, is highlighted in red. CpG sites that were linked to increased overall survival are highlighted in orange. CpGs with increased overall survival in our study highlighted in orange were chosen based on Random Forest analysis without imputed data

The results from the current study confirm, based on the ROC-curve analyses, that the mean methylation status of CpGs 79–82 in exon 1, as analyzed by the Therascreen kit (Qiagen) and commonly used to select patients for temozolomide administration, is reliably associated with response to temozolomide treatment and overall survival. In addition, our data suggest that methylation of other CpGs, particularly those located in the enhancer region of *MGMT*, merit special focus because of their strong correlation with overall survival (Fig. 6).

In particular CpG 86 (bp + 154), located in the enhancer region of *MGMT*, showed the highest predictor importance value in the Random Forest analysis, indicating a strong correlation with outcome. However, also other CpG sites, such as CpGs 75, 77, 78, 84, 88 and 96, demonstrated strong predictor importance scores in the Random Forest analysis, and are, thus, likely to be of prognostic value. Our findings are partially supported by previous studies (Fig. 6), however, these studies included some patients with *IDH*-mutant gliomas, which usually have a better prognosis, and are no longer classified as glioblastoma [15, 16]. Hegi et al. first reported a prognostic benefit in patients with *MGMT* methylated glioblastoma treated with temozolomide. She and her colleagues applied methylation specific PCR with the forward primer binding to CpGs 79–83 and the reverse primer binding almost entirely in the enhancer region, comprising the CpG sites 87–91 [5, 9]. Thus, only when both primers are bound to DNA, a PCR product can be detected. In a prospective study, Shah et al. reported that CpGs 42, 78, 84, 85, 86, 90 and 94 significantly correlated with mRNA expression, protein expression and progression free survival [23]. In addition, the study by Malley et al. discovered that methylation of CpGs 86, 89, 90 and 92 was critically correlated with protein expression [17]. Quilien et al. described that particularly CpGs 87 and 92 correlated with overall survival of glioma patients [20]. Among others, Bady et al. [1], who investigated the methylation pattern of glioblastomas based on 450 K analyses, found that CpGs 33 and 87 were the sites of most prognostic relevance, out of the 18 CpG sites that cover the CpG-island of *MGMT*. Notably, CpG 87 is also within the enhancer region and in proximity to the CpGs highlighted in the Random Forest analysis as being strongly correlated with overall survival (CpGs 86 and 88) (Fig. 3).

Although the data indicate a correlation between methylation of several CpG sites, there is some variation within each case across all tested CpGs. Therefore, our data further support the observation that the use of a combination of CpG sites might increase robustness against outliers. This has already been investigated by Chai et al. [3] and is used as a standard to determine the mean methylation level of four CpG sites with the help of the diagnostic pyrosequencing analysis kit Therascreen. Therefore we have also focused on a combination of four CpGs in our ROC-curve analysis. Even though there were no prominent differences observed based on our small cohorts, it might be refined in the future based on larger studies on glioblastomas.

In addition to *MGMT* methylation, our study further supports that temozolomide treatment and age of the patient are prognostically relevant markers [6]. In turn, there was no prominent association of tumor location with outcome of the patients. Other prognostic markers such as Karnofsky Performance Status or extend of resection were only partially available and have thus not been included in our analyses.

Interestingly, we observed two cases in the group of long-term survivors that demonstrated methylation of *MGMT* in the initial specimen, which was lost at recurrence (Additional file 3: Fig. S2). This finding is in line with the observations by Rabe et al. in vitro that there is a “Darwinian process with replacement of sensitive clones by resistant clones” under therapy [21]. Similar findings have also been described recently [11].

Therefore, reinvestigation of the methylation status of *MGMT* might be indicated in glioblastoma patients with prolonged survival to be able to estimate the therapeutic effect of temozolomide at recurrence.

The weaknesses of our study are partially inherited based on the scientific approach. Our aim was to focus on the outcome of glioblastoma patients and, thus, to search for DNA alterations of *MGMT* that are associated with survival. Therefore we selected cases based on outcome. This approach has the advantage that biologically relevant markers may be discovered, which might be masked in larger prospective studies due to comorbidities that may have an impact on outcome of the patients. In such case the biologically and prognostically relevant alterations of *MGMT* would no longer be perceivable as such. This explorative approach, however, is associated with drawbacks. First of all, the categorization of patients into long-term and short-term survivors, with discontinuous data due to the exclusion of patients who survived between one and three years, precluded us from using COX-regression models and Kaplan Meier curves to analyze survival. However, Random Forest regression highlighted prognostically relevant CpG sites regardless of confounding factors such as discontinuous survival data or missing values.

Still, the prominent predictor importance of CpG 86 was not reflected in the ROC analysis. The reason for this discrepancy is not entirely clear. Even though speculative, the following differences between both analyses might provide a plausible explanation:

Random Forest could be performed on the complete data set, including CpGs 23–74, as it is able to handle missing values. In contrast, both, ROC and Conditional Random Forest analyses required a complete data set, necessitating imputation of missing values. Still, also Conditional Random Forest analysis revealed a high predictor importance for CpG 86.

Both methods, ROC and Random Forest, take all methylation levels into account that have been determined using Sanger sequencing at each CpG site. However, since Random Forest is a multivariate analysis, it is able to reveal complex methylation patterns of several CpGs simultaneously, whereas for ROC analyses only the methylation level of a single CpG or a predefined combination of CpGs, such as, e.g., the mean methylation level of four predefined CpG sites, can be evaluated.

Another difference is that for ROC analyses the survival is dichotomized meaning that both, patients that survived slightly longer than 3 years fall into the same category as patients that survived 8 years. In fact, also patients that survived only 0.1 years and those who survived 0.9 years are matched in one category. Therefore, ROC analysis loses prognostic information, which is still present in the non-dichotomized Random Forest regression analysis. However, due to the study design there are no patients that survived between 1 and 3 years. Therefore survival time cannot be regarded as continuous variable and ROC analysis with a dichotomized survival data set is a valid investigational approach. Since our goal was to investigate multiple CpG sites that might be relevant for patient survival, especially with respect to long-term survival, we provided the survival in years to the Random Forest algorithm and used a regression based approach. Random Forest is considered to be robust against missing values, however, the influence of the discontinuous survival data in our cohort on the Random Forest result remains unclear, which necessitates further investigation in follow-up studies.

The data showed that CpG 86 is the site that best discriminates between long- and short-term survivors when comparing the true positive ratio (TPR), i.e., relative percentage of how many long-term survivors are methylated at the particular CpG site, and the false positive ratio (FPR), i.e., how many short-term survivors are methylated at the particular CpG site, at cut-off levels of 25, 30, 35 and 40% methylation. This result might partially explain, why Random Forest assigns more importance to this particular site (Additional file 5: Table ST1). As a side note, in clinical practice at the time of the investigation at the University Hospital in Zurich, Switzerland (USZ), a methylation level of 25% was considered to be most likely methylated, whereas values below 25% were considered to be likely unmethylated.

Since the cohort is rather small and there is no linear correlation between RF and ROC analysis, we are somewhat hesitant to conclude that CpG 86 represents the most important CpG site. However, the data are highly suggestive that the CpG sites of the enhancer region are especially relevant in terms of outcome. This observation is not only based on our study, but it has been demonstrated by others that this region is biologically and physiologically relevant (as pointed out in Fig. 6). We therefore maintain that this region needs additional scrutiny for further refinement of the *MGMT* methylation status of glioblastomas.

Furthermore, retrospective studies are hampered by well-known limitations such as selection and recall bias, among others. Accordingly, it is important to confirm the findings that emerge in larger prospective studies.

Pre-selection and comparison of rare cases, i.e. long-term survivors of glioblastoma, who are compared with patients, who died quickly from this disease, leads to unbalanced parameters between both cohorts. As expected, variation was noted in the patient cohort in terms of age and tumor location. Whereas median age at initial diagnosis in long-term survivors was about 50 years, the median age in short-term survivors was about 70 years. Furthermore, tumors of long-term survivors were often located in the frontal lobes, whereas the parietal regions were more frequently involved in short-term survivors. Results according to Random Forest analysis confirm the strong prognostic effect of age and temozolomide treatment, which had the strongest impact on overall survival, in line with current data [9, 14]. Although it is conceivable that larger tumor resections, which tend to be more feasible in the frontal lobes, could have influenced outcome, tumor location proved to be of minor importance in our Random Forest analysis.

Another confounder is that all patients of the long-term survivor cohort were treated with temozolomide, whereas only about half of the short-term survivor patients (11/24) received the drug. Whether or not temozolomide treatment might have improved overall survival in some of these patients remains unknown. However, due to poor general condition and early death, patients have been restricted from such treatment, and therefore, potentially relevant CpG sites might have been misinterpreted as being irrelevant for response to temozolomide and outcome.

Tissue samples from long term survivors were older and, thus, often associated with reduced DNA and RNA quality. Therefore we were unable to investigate mRNA expression for MGMT, which prevented us from directly correlating CpG-methylation and MGMT-expression and comparing our data with the results of Shah et al. [23]. The reduced DNA quality resulted in the exclusion of 5 long-term survivors and 1 short term survivor as well as several missing values, which needed to be imputed for additional Conditional Random Forest analyses. However, focusing on the area covered by the ATG1 primer pair, which generated almost a complete data set with only 2 missing values at 1377 sites (0.15%), bypassed this problem to a large extent. This enabled us to concentrate on this region and to draw conclusions, due to valuable comparison between almost all CpGs within this area.

Finally, WHO 2021 diagnostic criteria could not be applied due to inadequate tissue samples for additional studies. In other words, tumor classification was based on the CNS WHO (2016) criteria as no molecular data other than IDH-status were available for further molecular classification of the tissue samples.

## Conclusion

Our retrospective study confirms differences in age and *MGMT* methylation pattern between long-term and short-term survivors of glioblastoma. We found that long-term survivors are more often younger with methylated tumors, whereas short-term survivors were mainly older and demonstrated low *MGMT* methylation levels [8, 12]. In terms of prognostically relevant CpG sites, the study provides further evidence that the methylation status of the commonly used combination of CpGs 79–82, as analyzed by the Therascreen MGMT pyro kit, is of prognostic value. However, our data also suggest that other CpG sites and combinations of CpG sites have a similar or even stronger correlation with overall survival and possibly response to temozolomide treatment. As shown in other studies [17, 23], we observed that the enhancer region merits special focus. Particularly CpG 86 (bp + 154) was of interest in our cohort as it demonstrated remarkably high values in the Random Forest regression analysis, and is therefore likely to be associated with prognosis.

Our findings should be confirmed in larger prospective studies of glioblastoma patients in order to draw definitive conclusions that could lead to both a more refined analysis of *MGMT* with potentially resulting stratification of temozolomide administration, as well as better prediction of the prognosis of patients suffering from this devastating disease.

### Supplementary Information

**Additional file 1.** Supplementary materials and methods

**Additional file 2: Fig. S1. **Missing values.

**Additional file 3: Fig. S2. **CpG methylation of MGMT can be lost over time.

**Additional file 4: Fig. S3. **Correlation matrix of the methylation data (CpGs 75-101) from all patients, as well as age and survival.

**Additional file 5: Fig. S4. **ROC results for single CpGs.

**Additional file 6: Table S1. **Individual characteristics of patients are listed under patient characteristics. Methylation results per sample and CpG site are given under CpG-methylation results. Here, the individual CpG sites were listed according to the primers that were used to detect the CpG site, the CpG-number used in our study, the relative position according to the transcription start side, as defined by Nakagawachi et al.(Nakagawachi et al., 2003), and the position according to the UCSC Browser on Human Feb. 2009 (GRCh37/hg19) Assembly. The lowest CpG-methylation was set to 5. CpG sites were no results could be detected are highlighted as “NaN”. Additionally, “NaN”s that were imputed using the MissForest algorithm and rounded up to integers are highlighted in red. The TPR and FPR per methylation cut-off value and CpG site, as well as the TPR-FPR value is shown. Maximum values of TPR-FPR are highlighted in yellow.

## Data Availability

The dataset supporting the conclusions of this article are included within the article and its additional files.

## Abbreviations

*MGMT*: O6-*methylguanine*-DNA *methyltransferase*
*IDH*: *Isocitrate dehydrogenase*
Bp: Base pair
OS: Overall survival
PCR: Polymerase chain reaction
MSE: Mean squared error
OOB-Data: Out of bag-data
SST: Sum of squares total
AUC: Area under the curve
ROC-curve: Receiver operating characteristic curve
SEM: Standard error of the mean
TPR: True positive rate
FPR: False positive rate
