# Supplementary material for: MGMT methylation pattern of long-term and short-term survivors of glioblastoma reveals CpGs of the enhancer region to be of high prognostic value
Source: Acta Neuropathol Commun. 2023 Aug 28;11:139. doi: 10.1186/s40478-023-01622-w (PMC10463744; doi:10.1186/s40478-023-01622-w)
Supplement: Supplementary file 1 — Additional file 1. Supplementary materials and methods [file 40478_2023_1622_MOESM1_ESM.docx]

Supplementary Material and Methods

DNA was extracted from FFPE material or, if available, from frozen material. Only samples with a tumour cell content > 50% were included. The extracted DNA was purified using the QIAamp DNA Mini Kit (Qiagen) according to the manufacturer’s protocol. DNA was converted using the Qiagen EpiTect^®^ Bisulfite Kit. For subsequent Sanger sequencing of the *MGMT* region of interest, we designed three primer pairs (Prom, E1I1 and ATG1) (table 1) covering 79 CpG-sites ranging from chr10:131,265,101-131,265,710 (UCSC Browser on Human Feb. 2009 (GRCh37/hg19) Assembly).

| Primer name | Fwd/rev | Nucleotide sequence 5´-3´ |
| --- | --- | --- |
| Prom | Fwd | TGT AAA ACG ACG GCC AGT *TCC CTC CTC T*TT ATT TTT GTG ATA GGA AAA GG |
|  | Rev1 | CAG GAA ACA GCT ATG ACC *AAG AGG AAG GG*A AAA CCT AAA AAA AAC AAA AAA AC |
|  | Rev2 | CAG GAA ACA GCT ATG ACC *AAG AGG AAG GG*A AAA CCT AAA AAA AAC TAA AAA AC |
| E1/I1 | Fwd1 | TGT AAA ACG ACG GCC AGT *TCC CTC CTC T*GT TTT TTT GTT TTT TTT AGG TT |
|  | Fwd2 | TGT AAA ACG ACG GCC AGT *TCC CTC CTC TG*T TTT TTA GTT TTT TTT AGG TT |
|  | Rev | CAG GAA ACA GCT ATG ACC *AAG AGG AAG GG*C TAA ACA ACA CCT AAA AAA CAC TT |
| ATG1 | Fwd | TGT AAA ACG ACG GCC AGT *TCT CCT CTA* GGA TAT GTT GGG ATA GTT |
|  | Rev | CAG GAA ACA GCT ATG ACC AAG AGG AAG *GGC* TAA ACA ACA CCT AAA AAA CAC TT |
| M13 | Fwd | GTA AAA CGA CGG CCA GT |
|  | Rev | CAG GAA ACA GCT ATG ACC |
| Re-Prom |  | GTA GAT TGT TTT AGG TT |
| Re-E1I1 |  | GAT TTG GTG AGT GTT TG |

PCR cycling conditions consisted of initial denaturation (95°C, 5 min), followed by 40 cycles of denaturation at 95°C for 1 min, primer annealing at 52°C for 1 min, and extension at 72°C for 5 min with a final extension step of 10 min at 72°C. The PCR reactions were done in a final volume of 50 μl (1 x PCR reaction buffer, 5 mM MgCl_2_ , 0.2 mM of each of the four primers).

Specific amplicons were purified using the Qiagen MinElute Gel Extraction Kit. Purified PCR products were sequenced using M13fwd and M13rev primers on an ABI 3100 genetic analyzer using Big Dye terminator chemistry (1:1 mixture of ABI PRISM^®^ BigDye^TM^ Terminator v.3.1 Cycle Sequencing Kit; Applied Biosystems). To allow correct determination of methylation data for the entire sequence, Prom and E1/I1 PCR-products were re-sequenced using the correspondent sequencing primer Re-Prom and Re-E1I1 (table1). The methylation percentage of CpG-sites, ranging from CpG 23 (bp -300) to CpG 101 (bp +309) of *MGMT*, in the tumour tissue was evaluated using the Mutation Surveyor software (softgenetics). Bisulfite converted tonsil-DNA served as reference sequence.

Absolute values were used for further statistical analyses.

*IDH* sequencing

The primers for the detection of *IDH1/2* sequence alterations are listed in the table S2. The PCR conditions were 95°C for 5 min (1 cycle), followed by 40 cycles of 95°C for 1 min, 55°C for 1 min and 72°C for 1 min, and finally extension at 72°C for 10 min with AmpliTaq™ 360 DNA Polymerase (Applied Biosystems).

| Gene | Reaction ***** | Name | Primer Sequence (5’ 🡪 3’) |
| --- | --- | --- | --- |
| *IDH1* | long | IDH1_3rd up B  IDH1_2nd low | GAGCTCTATATGCCATCACT  AAAACATGCAAAATCACATTAT |
|  | short | IDH1_short up  IDH1_2nd low | AATGGCACCATACGAAAT  AAAACATGCAAAATCACATTAT |
| *IDH2* | long | IDH2_long up  IDH2_long low | CAAATTCTGGTTGAAAGATGGCG  AAGAGGATGGCTAGGCGAGGA |
|  | short | IDH2_short up  IDH2_short low | GGAGCCCATCATCTG  AAGTCTGTGGCCTTGTACT |
