# Supplementary figures and images for: MGMT methylation pattern of long-term and short-term survivors of glioblastoma reveals CpGs of the enhancer region to be of high prognostic value
Source: Acta Neuropathol Commun. 2023 Aug 28;11:139. doi: 10.1186/s40478-023-01622-w (PMC10463744; doi:10.1186/s40478-023-01622-w)

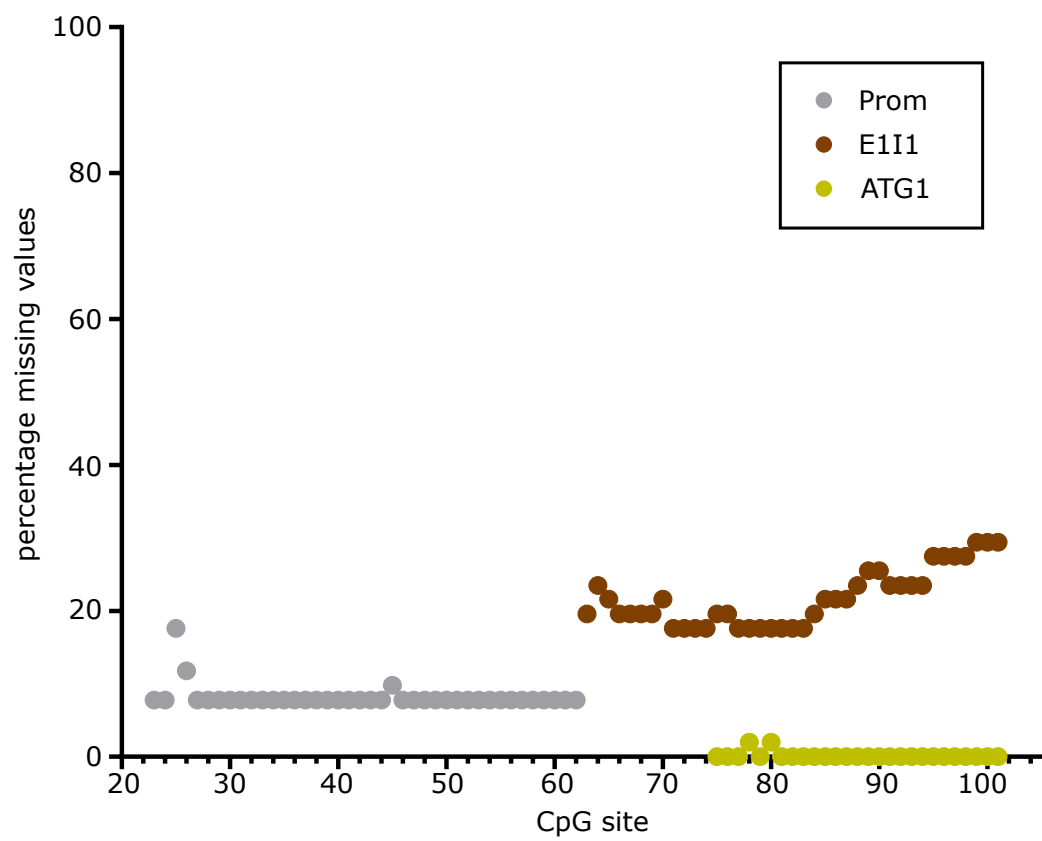

Supplement: Supplementary file 2 — Additional file 2: Fig. S1. Missing values. [file 40478_2023_1622_MOESM2_ESM.pdf]

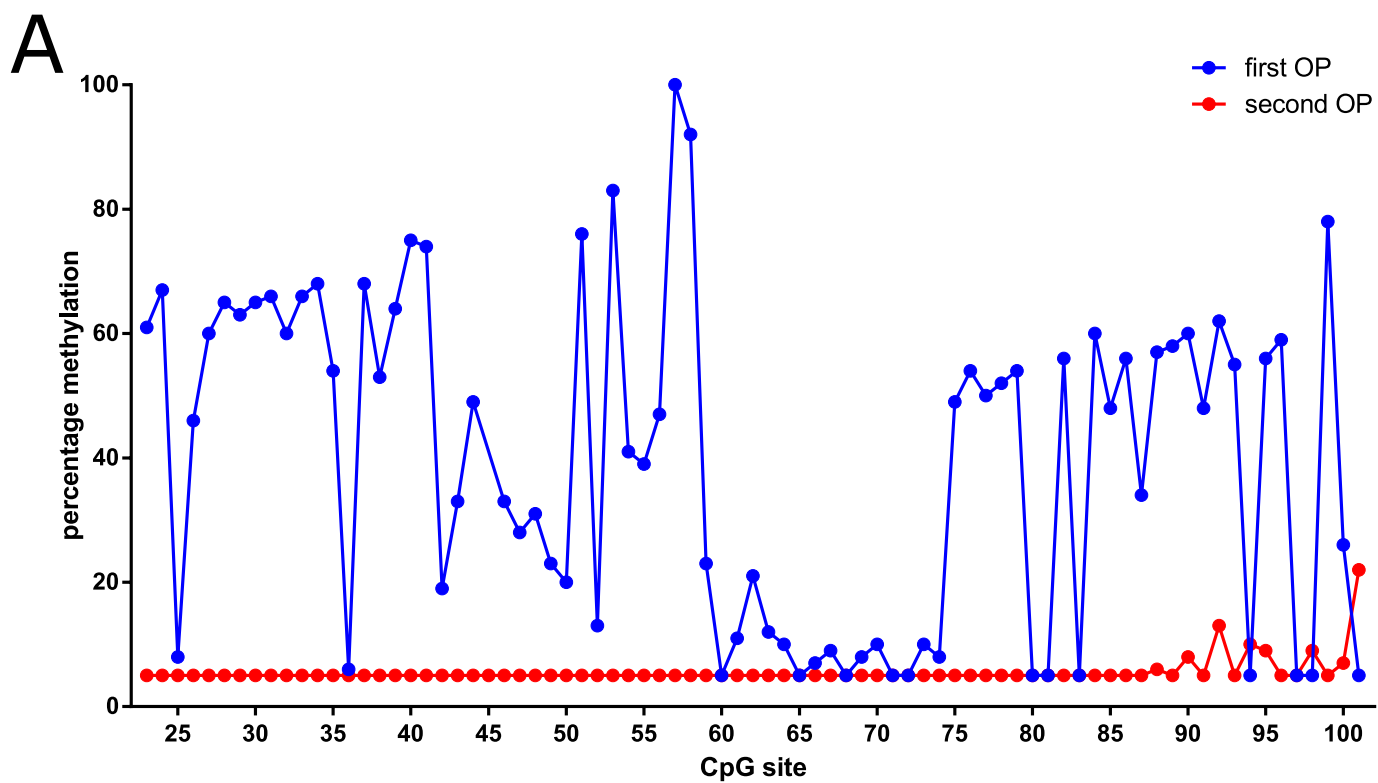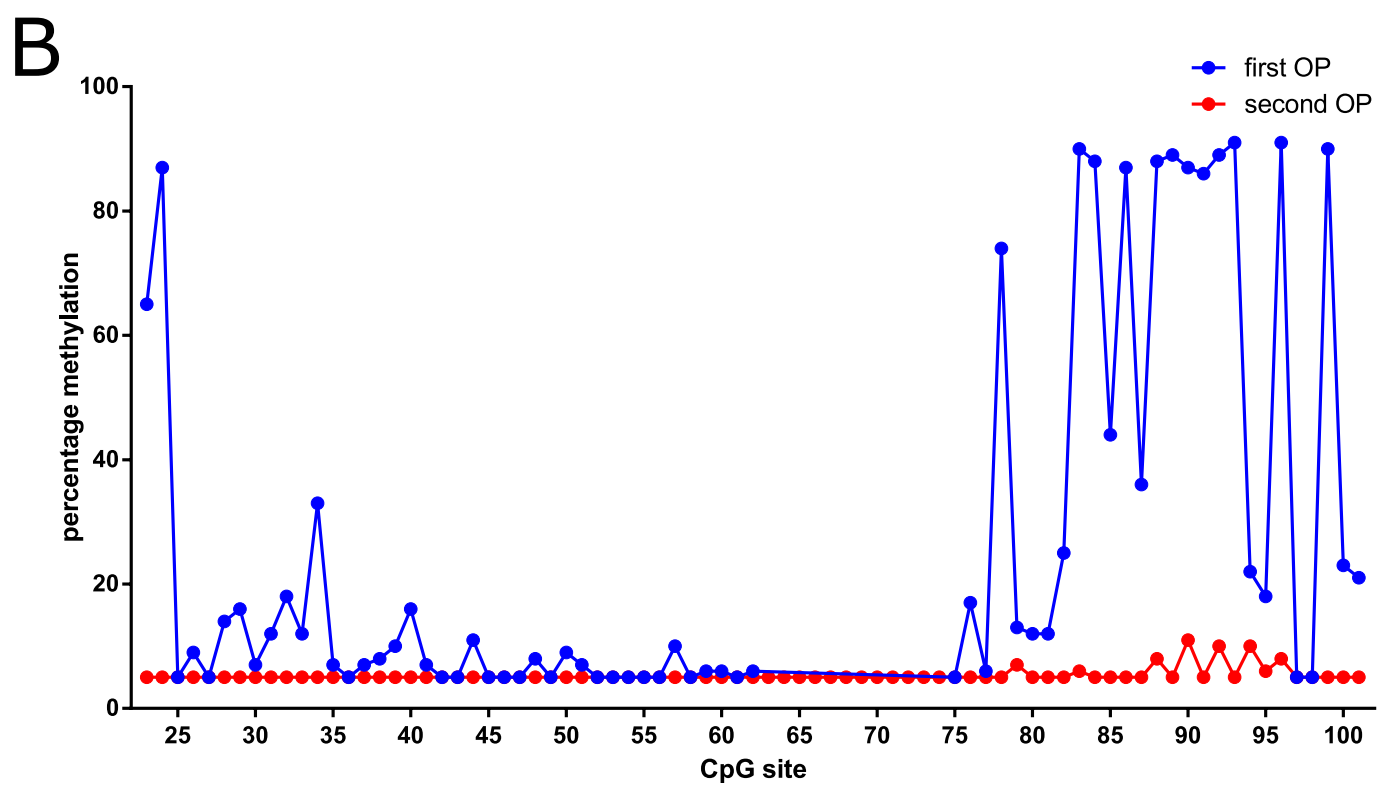

Supplement: Supplementary file 3 — Additional file 3: Fig. S2. CpG methylation of MGMT can be lost over time. [file 40478_2023_1622_MOESM3_ESM.pdf]

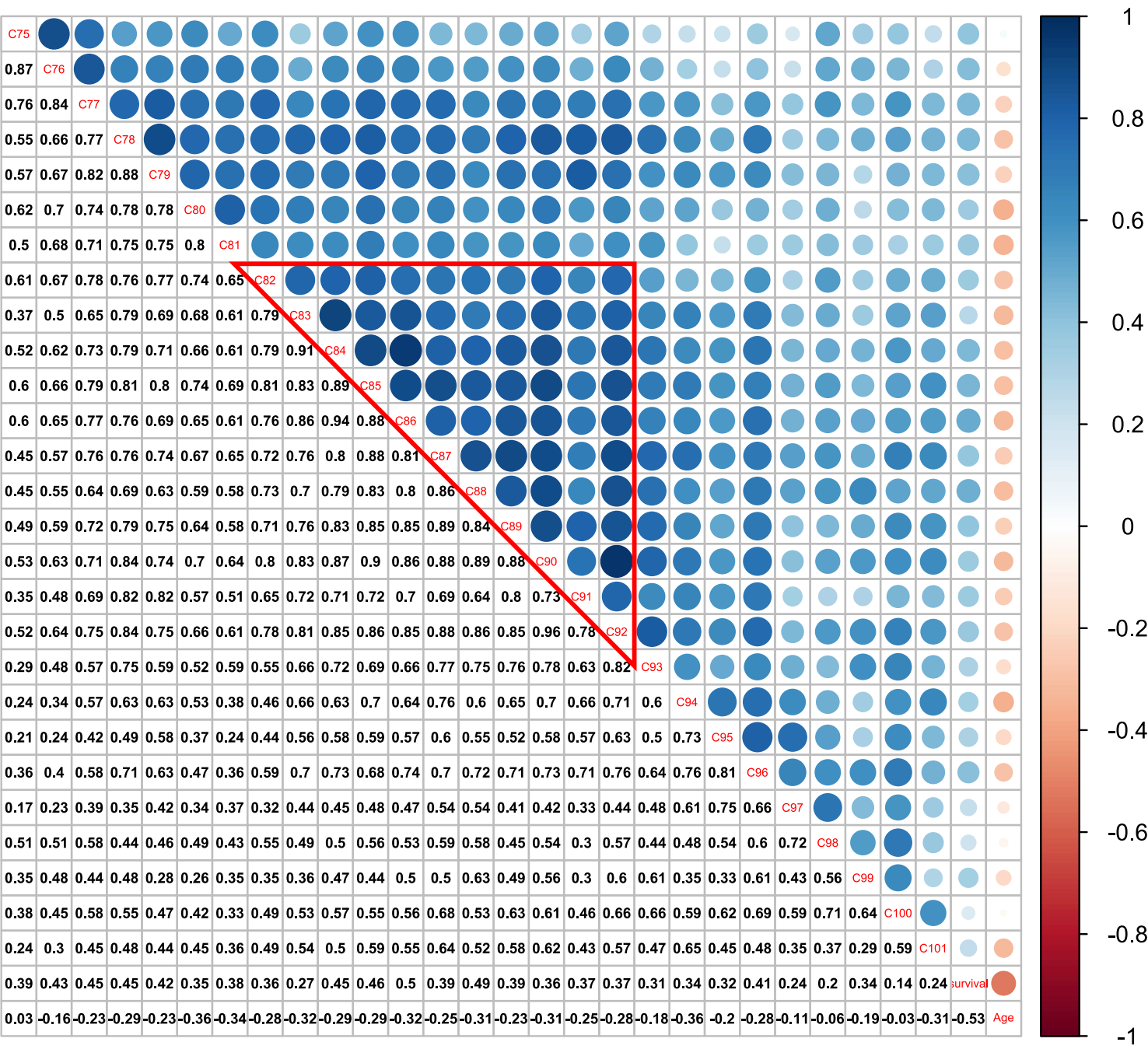

Supplement: Supplementary file 4 — Additional file 4: Fig. S3. Correlation matrix of the methylation data (CpGs 75-101) from all patients, as well as age and survival. [file 40478_2023_1622_MOESM4_ESM.pdf]

# A

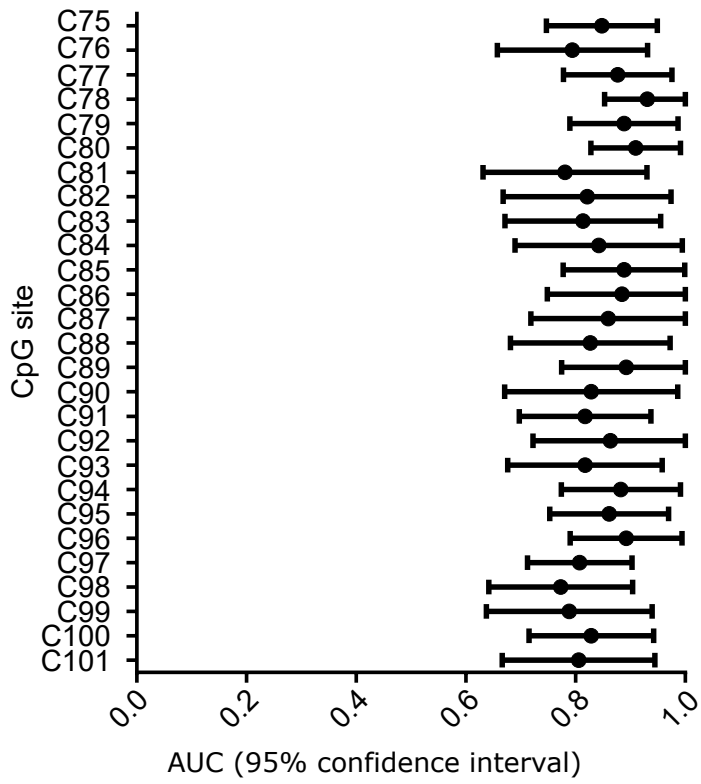

# B

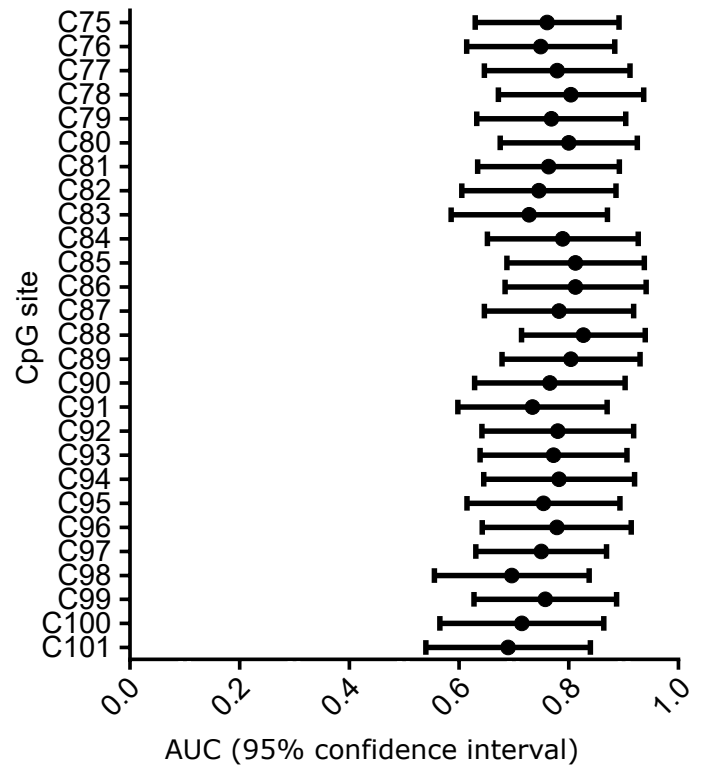

Supplement: Supplementary file 5 — Additional file 5: Fig. S4. ROC results for single CpGs. [file 40478_2023_1622_MOESM5_ESM.pdf]
